# Retrosplenial Cortex and Long-Term Memory: Molecules to Behavior

**DOI:** 10.1155/2015/414173

**Published:** 2015-08-25

**Authors:** Travis P. Todd, David J. Bucci

**Affiliations:** Department of Psychological and Brain Sciences, Dartmouth College, Hanover 03755, NH, USA

## Abstract

The retrosplenial cortex (RSC) is reciprocally connected with the hippocampus and various parahippocampal cortical regions, suggesting that RSC is well-positioned to contribute to hippocampal-dependent memory. Consistent with this, substantial behavioral evidence indicates that RSC is essential for consolidating and/or retrieving contextual and spatial memories. In addition, there is growing evidence that RSC neurons undergo activity-dependent plastic changes during memory formation and retrieval. In this paper we review both the behavioral and cellular/molecular data and posit that the RSC has a particularly important role in the storage and retrieval of spatial and contextual memories perhaps due its involvement in binding together multiple cues in the environment. We identify remaining questions and avenues for future research that take advantage of emerging methods to selectively manipulate RSC neurons both spatially and temporally and to image the RSC in awake, behaving animals.

## 1. Introduction

The retrosplenial cortex (RSC) is positioned at the interface between sensory cortical regions and the myriad of structures that compose the parahippocampal-hippocampal memory network. Importantly, the connections between RSC and these structures are reciprocal (i.e., afferent and efferent), suggesting that RSC not only contributes incoming sensory information to the hippocampus, but may also serve as a critical site of information storage.

In this paper we consider the cellular and behavioral evidence that supports the involvement of RSC in spatial and contextual memory. We begin by considering the functional neuroanatomy of RSC and provide a working model of the nature of information processing within RSC during learning and memory. We then discuss the results of lesion and inactivation studies that demonstrate the involvement of RSC in the consolidation and/or retrieval of spatial and contextual memories. This is followed by a review of recent studies of the contribution of RSC to the extinction of fear memory. Next, we turn to evidence of memory-related changes in neuronal function (i.e., neural plasticity) in RSC. Finally, we conclude by positing a specific role for RSC in long-term memory and suggest avenues for future research. Studies using laboratory rodents are emphasized because the bulk of the research on plasticity molecules and manipulations of RSC has been carried out in rats. Additionally, other recent reviews have aptly considered the findings from neuroimaging and other approaches used to study RSC in primates [1, 2].

## 2. Connectivity of the Retrosplenial Cortex

**Figure 1:**
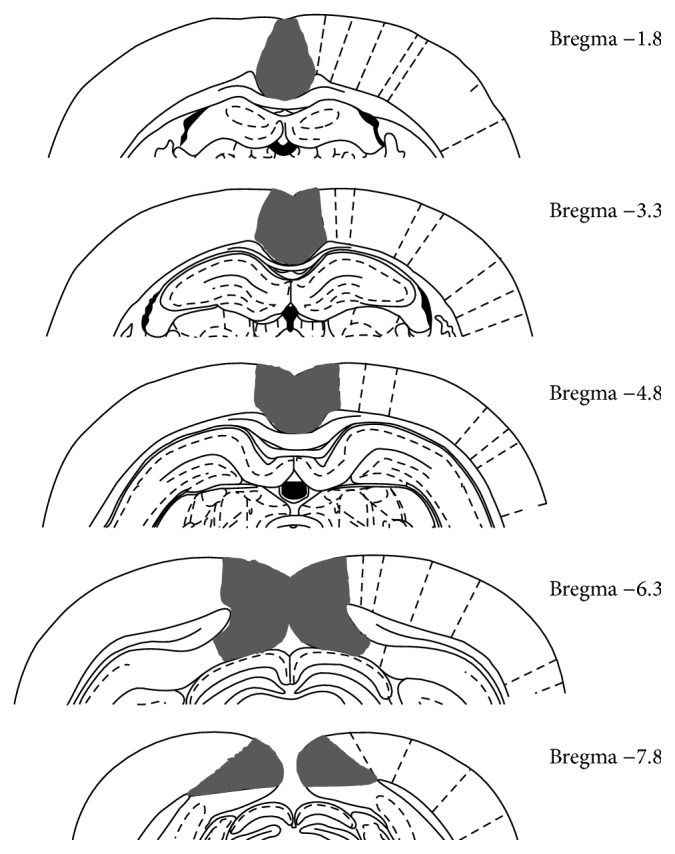
Schematic diagram illustrating the rostrocaudal extent of the RSC (black fill) in rats. Adapted from Paxinos and Watson [91].

**Figure 2:**
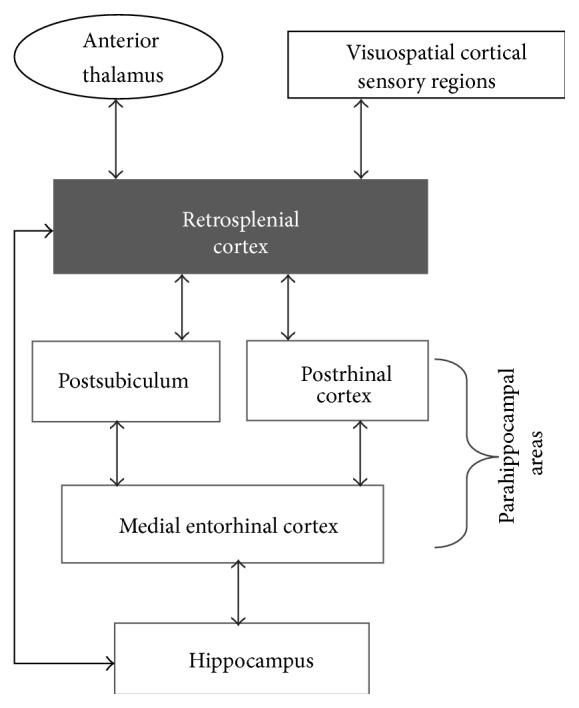
Major cortical and thalamic afferents and efferents of RSC. Only the densest interconnections are illustrated for simplicity.

The RSC is a relatively large, polymodal midline structure that extends ~8 mm along the rostrocaudal axis of the rat brain (Figure 1). Among its various connections, RSC receives input from visuospatial cortical sensory areas and has strong reciprocal connections with visual cortex (areas 17 and 18b), cingulate cortex, and multiple parahippocampal regions (postrhinal cortex, medial entorhinal cortex, and the postsubiculum) as well as the hippocampus itself [3–11], as show in Figure 2. The thalamic connections of RSC include both afferent and efferent connections with anterior and lateral thalamic nuclei [12, 13], structures that are involved in processing spatial information [14]. Thus, RSC is suited as a sensory integration center [7, 8, 15] located at the interface between visuospatial cortical and thalamic regions and structures composing the hippocampal memory system [9–11, 16].

## 3. What Is the Functional Contribution of RSC to Learning and Memory?

The pattern of connectivity between RSC and hippocampal-parahippocampal structures suggests that RSC is well-positioned to participate in hippocampal-dependent functions. Consistent with this, a growing body of evidence indicates that RSC is a critical component of the so-called “*where/when*” pathway (Figure 2), a network of cortical structures (which also includes the medial entorhinal cortex, postrhinal cortex, and visuospatial regions) that provides the hippocampus with information regarding the physical and temporal context in which an object/event occurs [2, 17–22]. Processing contextual information involves several different components, such as encoding and forming associations between the neutral sensory stimuli that compose an environment, ascribing behavioral significance to those associations (e.g., pairing with reinforcers), and updating stored associations to account for new information (e.g., [23]). Based on findings from prior behavioral studies [24–36], we and others have proposed that a specific contribution of RSC to processing where/when information may be forming and storing the associations among the various sensory stimuli that are present in a learning environment [1, 7, 37, 38]. Furthermore, the extant data indicate that the role of RSC in this process is not restricted only to physical stimuli but also includes temporal stimuli [39] that can also define the context in which an object or event is experienced [40–43]. Conversely, consistent with its specific role in the* where/when* circuit, RSC is not necessary for forming an association between an individual conditioned stimulus (CS) and an outcome (e.g., tone and shock [27, 28], c.f., [31]), which instead is processed by a somewhat separate cortical circuit (the “*what*” pathway consisting of regions including perirhinal cortex and lateral entorhinal cortex [44]).

We recently tested the involvement of RSC in forming associations between neutral sensory stimuli using a behavioral paradigm, sensory preconditioning, which isolates the formation of stimulus-stimulus associations from pairing those stimuli with a reinforcer. During the preconditioning phase of this procedure, rats were presented with serial pairings of a tone and a light (each cue presentation was 5 sec in duration). On a subset of trials in each session another auditory cue (white noise) was presented by itself. Because no reinforcement was delivered during this phase, rats had the opportunity to form an association between the tone and light in the absence of any biologically significant outcome. During the subsequent conditioning phase the light was paired with a food reward. In a final test phase, the tone and the white noise were presented alone on intermixed trials. Normal rats exhibited more conditioned responding to the preconditioned auditory cue (the tone) compared to the unpaired cue (white noise) during the test session, reflecting the formation of a stimulus-stimulus association between the tone and the light during the preconditioning session (i.e., sensory preconditioning [45–47]). In contrast, silencing RSC neurons during the preconditioning phase eliminated the sensory preconditioning effect, suggesting that the RSC is needed for forming the stimulus-stimulus association between the tone and the light [48].

Interestingly, it has recently been shown that the hippocampus is not active [49] nor necessary [50] for forming the initial stimulus-stimulus associations in similar sensory preconditioning paradigms. Together with the findings of Robinson et al. [48], this supports the intriguing idea that another region(s), such as RSC, forms the stimulus-stimulus associations that the hippocampus then uses to form contextual and spatial representations which are subsequently incorporated into existing schemas [7, 19, 34, 37, 38]. Rigorous testing of this idea awaits future study. Moreover, because the sensory preconditioning procedures described above used discrete and phasic stimuli like tones and lights, caution should be exercised in drawing comparisons to the formation of associations between sensory stimuli that are static background cues, like those that compose a physical context. That said, the process of forming stimulus-stimulus associations is critical for learning about contexts and studies of sensory preconditioning may thus inform how animals learn about contexts.

## 4. Involvement of RSC in Memory Storage and Retrieval

### 4.1. Contextual Fear Memory

In experiments with laboratory rodents,* contexts* are usually defined as the chambers in which conditioning occurs and are composed of various visual, tactile, and olfactory characteristics. In contextual fear conditioning experiments, rats will exhibit freezing behavior (immobility except for respiration) when they are returned to a context where they have previously received a mild foot shock [51], indicating that contexts can directly elicit conditioned fear responses [42].

Evidence from lesion and inactivation studies suggests that the RSC is necessary for recalling contextual memories. Indeed, studies using contextual fear memory paradigms have demonstrated that RSC is involved in postencoding processes, such as the consolidation, storage, and/or retrieval of previously formed associations between stimuli in the environment. For example, permanent lesions of the RSC carried out one day after training produce dramatic reductions in freezing behavior when the rats are placed back in the training chamber after recovering from surgery [27]. Importantly, in the same study, RSC lesions had no effect on fear conditioning to a discrete, Pavlovian CS (e.g., tone-shock pairings, c.f., [31]). These findings indicate that RSC is necessary particularly for the retrieval of* contextual* fear memory. Interestingly, damage to RSC that takes place after training has a greater effect on the retrieval of contextual fear memory than lesions carried out prior to training. One interpretation of this pattern of results is that in the absence of a functioning RSC (i.e., pretraining lesion), other brain systems or strategies may be able to at least partially compensate during the encoding and/or retrieval of contextual fear. However, in the intact brain, RSC may be part of the preferred circuitry for processing contextual information. Thus, damage to RSC after training may have a more dramatic effect on the retrieval of context fear memory since memory formation was reliant on RSC.

Other studies have shown that NMDA glutamate receptors in RSC are necessary for the retrieval of contextual fear memories. For example, infusion of APV, an antagonist of NMDA glutamate receptors, into the RSC prior to a memory retrieval session reduces freezing to the context in which conditioning had occurred either 1 day or 36 days earlier, indicating that NMDA glutamate receptors in the RSC are integral for the retrieval of contextual fear [52]. Moreover, this effect was mimicked by specifically blocking NMDA receptors that contain the NR2A subunit; administration of an NR2B-selective antagonist was without effect. There was also no effect of blocking AMPA glutamate receptors. Consistent with the lesion data [27], blocking NMDA glutamate receptors in RSC had no effect on the retrieval of fear conditioned to a discrete, Pavlovian CS. Thus, the role of the RSC in fear memory appears to be specific to the expression of* contextual* fear memory.

Inhibitory avoidance is another behavioral paradigm that involves learning and recalling that an aversive stimulus was paired with a specific environment. In a typical inhibitory avoidance task, rats are placed in the lighted side of a two-compartment apparatus and voluntarily enter the darkened compartment within a few seconds, since rats have a natural aversion to brightly lit areas. Upon entry into the dark compartment a mild foot shock is delivered. Memory retrieval is assessed by returning the rat to the lighted side at a later time and measuring the latency to cross over into the dark compartment. Intact rats avoid the dark compartment and typically enter it only after a long period of time (e.g., several minutes), indicative of the memory that it had previously been paired with shock. Inhibitory avoidance memory is impaired by temporary inactivation of neurons in the RSC at the time of retrieval as evidenced by a reduction in the latency to enter the dark compartment [53]. These data complement those from studies of contextual fear conditioning and support the involvement of RSC in recalling memories for contexts.

### 4.2. Spatial Memory

Various studies using spatial memory paradigms have also demonstrated that RSC is involved in the consolidation, storage, and/or retrieval of associations between stimuli in the environment. In the case of spatial memory, organisms must learn and remember* where* reinforcement is located in the environment so that they can successfully navigate to the item [54]. For example, in the radial arm maze rats must learn which of the arms contains a food item, and in the Morris water maze rats must learn where a hidden escape platform is located in a pool of opaque water. In both cases, rats learn about and use cues in the environment to guide their behavior. Importantly, spatial memory, like contextual fear memory, relies upon the binding of stimuli in the environment to form cohesive, conjunctive representations [55, 56].

Several lines of evidence indicate that RSC has an integral role in spatial memory [55, 57]. For example, temporary inactivation of RSC neurons at the time of retrieval impairs performance in the water maze [58]. Other experiments demonstrate that permanent lesions of RSC carried out after training produce retrograde amnesia for spatial memories [59]. For instance, lesioning RSC either 1 day or 4 weeks after training produces deficits when memory is subsequently tested after recovery from surgery. These findings indicate that RSC is critical for retrieving spatial memories. Interestingly, it has been shown that lesions of RSC carried out prior to training also produce deficits in spatial memory, particularly at longer training-to-testing intervals [59].

In summary, the use of permanent lesion or temporary inactivation techniques indicates that RSC is necessary for the consolidation, storage, and/or retrieval of contextual and spatial memories. Additional studies that differentiate between the involvement of RSC in memory consolidation, storage, and retrieval are needed to pinpoint the specific timeframe of RSC involvement in contextual memory. Nevertheless, the findings to date support the hypothesis that RSC is a potential site of long-term storage of spatial and contextual memory [60], perhaps due to the RSC's involvement in binding together multiple cues in the environment [1].

## 5. Extinction Learning and Memory

The evidence described above supports a role for the RSC in the retrieval of spatial as well as contextual fear memories. Perhaps equally important to the understanding of RSC plasticity in learning and memory is the role of RSC in* extinction*, a fundamental behavior change process. In extinction, repeated presentation of the previously conditioned cue or context, in the absence of the reinforcer (i.e., footshock in fear conditioning paradigms), results in a decrease in the conditioned response (see [41, 61]). Extinction learning is essential for the survival or organisms, because it allows them to adapt to changes in their environment [53]. Just as RSC has a role in the retrieval of contextual fear memories it also has a role in the extinction of contextual fear. For example, NR2B subunit-containing NMDA receptors are necessary for the extinction of older (i.e., remote) but not more recent contextual fear (e.g., [62]). This indicates that, at least for extinction of contextual fear, the role of the RSC is dependent upon the age of the memory.

The loss of behavior observed in extinction does not reflect unlearning or erasure of the original memory [41, 61]. Instead, it is now widely understood that extinction results in new learning that is at least partly dependent on context [63]. For example, responding to an extinguished Pavlovian CS will return when that CS is tested outside of the context of extinction (e.g., [64]). The fact that extinction of Pavlovian CSs is controlled by the context suggests an important role for the RSC, which has already been shown to be crucial for contextual learning and memory. In fact, there is recent evidence that extinction of a trace CS engages the RSC (in trace conditioning procedures, a short time interval is inserted between CS offset and US onset. This is in contrast to delay conditioning procedures, where CS offset coincides with US onset). For example, infusion of APV into anterior RSC during trace extinction impairs extinction retention when tested 1 day later. Thus, plasticity in the RSC is necessary for the extinction of fear to a trace CS [65].

The fact that RSC contributes to extinction of trace CS memories is especially interesting considering RSC appears to have no influence on retrieval of delay CS memories [27, 52], c.f. [31] or the extinction of delay CS memories [65]. However, it remains to be determined why trace, but not delay, extinction relies upon the RSC [65]. For example, if the RSC is simply providing contextual information during extinction learning, then one would expect both delay and trace extinction to involve RSC. Kwapis et al. [65] have instead suggested that the dissociation of RSC involvement in delay versus trace extinction might be due to trace memories, and not delay memories, being initially stored in the RSC. The additional assumption is that extinction learning engages synapses that support the original memory. However, as Kwapis et al. [65] state, it remains to be determined if the original trace CS memory is stored in the RSC.

## 6. Activity-Dependent Neural Plasticity in RSC

The data described thus far indicate that RSC may be a site of long-term storage for contextual and spatial memory. If so, then RSC would be expected to exhibit cellular and physiological signatures of memory formation and storage. Indeed, a multitude of activity-dependent signaling molecules and mechanisms have been linked to the formation of long-term memory, including the activation of transcription factors, protein synthesis, dendritic growth and branching, and the induction of long-term potentiation (LTP) and depression (LTD).

Consistent with this notion, RSC neurons are known to possess a variety of intracellular molecules that have been associated with activity-related plasticity, including various transcription factors (e.g., Fos, Zif268, Arc) and growth factors (e.g., BDNF). In addition, an extensive line of research shows that the expression of transcriptions factors and growth factors in RSC can be altered following disconnection from structures known to be involved in memory processing. For example, lesions of the hippocampus significantly reduce the expression of Fos and Zif268 in RSC neurons [66], suggesting that projections from hippocampus to RSC modulate the expression of genes involved in synaptic plasticity and memory formation. Similarly, damage to anterior thalamic nuclei reduces expression of the same genes, as well as expression of other genes that have been shown to be involved in neuroplasticity, such as CREB, neuritin1, ncs-1, 5htrc, and kcnab2, and in genes involved in cell-signaling (e.g., scamp1, neurexin1, and exoc7 [67–70]). Conversely, stimulation of thalamic input to RSC resulted in increased Fos expression [71]. Importantly, the changes in gene expression following denervation of RSC did not result in significant atrophy of RSC neurons [66] but were likely due to changes in the amount of activity that RSC neurons could sustain [72]. Thus, the alterations in transcription factor and growth factor expression reflect a more subtle alteration in function within RSC. Furthermore, none of the changes observed following hippocampal or anterior thalamic lesions were observed when other structures, such as the entorhinal cortex or postrhinal cortex, were damaged [66, 70]. Together, these findings indicate that plasticity-related molecules are expressed by RSC neurons and that interactions between RSC and the hippocampus and the anterior thalamus may be particularly important in promoting activity-related plasticity in RSC.

In addition to changes in gene expression associated with plasticity and cell signaling, other studies have shown that damage to the anterior thalamus disrupts LTD [73] and reduces neural excitability [74] of RSC neurons. Changes in synaptic activity, including LTD and LTP are thought to be fundamental substrates underlying learning and memory formation [75]. Thus, disruptions in the ability of RSC neurons to undergo synaptic modification may contribute to impairments in long-term memory formation and/or retrieval. Relatedly, anterior thalamus lesions [76] or excessive neural excitation (status epilepticus) [77] cause a reduction in dendritic spine density in RSC and decrease the expression of the BDNF receptor, TrkB [77]. Dendritic restructuring and alterations in BDNF signaling are likewise thought to be critically important to memory formation [78, 79]. Together, these findings indicate that RSC neurons exhibit activity-dependent changes in the expression of a variety of genes associated with neural plasticity, as well as alterations in dendritic structure and physiological manifestations of plasticity (e.g., LTD/LTP). As described in the following section, recent studies have extended this research to investigate neural plasticity in RSC specifically during memory formation and recall.

## 7. Memory-Related Plasticity in RSC

Demonstrating that RSC neurons contain the machinery to undergo activity-dependent plasticity is informative and suggestive that RSC has the potential to undergo the plastic changes traditionally thought to underlie memory. However, it is crucial to determine whether those mechanisms are actually at work during memory formation and retrieval. Several recent studies have now addressed this by assessing the activation of RSC neurons at different stages of memory retrieval. For example, immediate early genes such as H1a are expressed in RSC following training in the water maze [37], and metabolic activity is increased in RSC at 24 and 48 hours after training [72]. In addition, after rats were trained in a five-arm spatial maze, expression of Fos and Zif268 was examined during a single retention trial either 1 day or 30 days after training [80]. Interestingly, RSC neurons expressed more Zif268-positive cells during the 30-day retention test compared to the 1-day test. Other memory-related molecules, such as Arc, are elevated during spatial memory tests at both 1 day and 30 days after training in a water maze task [81]. The reasons for the differential expression of these molecules at different times after training remain to be elucidated, but importantly, in both studies, the expression of these transcription factors decreased over time in other brain areas (e.g., hippocampus, posterior cingulate cortex), indicating that RSC is among a select set of cortical regions that exhibit significant neural activity when memories are retrieved long after training.

It is well established that de novo protein synthesis in hippocampus and other structures is required for many forms of long-term memory [82]. Similarly, it has recently been shown that protein synthesis in the RSC is necessary for the consolidation of fear memories [83]. For example, infusion of a protein synthesis inhibitor into anterior RSC fifteen minutes prior to inhibitory avoidance training impaired memory retrieval at tests either 2 or 7 days later. This indicates that protein synthesis in RSC during or shortly after training is important for the formation of inhibitory avoidance memory. In contrast, infusing the protein synthesis inhibitor 12 hours* after* training produced retrieval deficits at the 7th day, but not the 2nd day retention test [84]. This finding suggests that protein synthesis in RSC at longer times after training is necessary for the formation of long-lasting memory. This is consistent with theories that posit a role for hippocampus primarily in recalling recent contextual and spatial memories, while a network of cortical regions is responsible for longer term storage (remote memory).

## 8. Current Research Directions and Avenues for Future Studies

Recently, new technologies such as optogenetics and genetic tagging methods have been brought to bear on questions relating to the involvement of RSC in memory. In one study, a c-fos genetic tagging approach was used to label cells that were active during contextual fear conditioning [85]. When tagged cells in RSC where later reactivated optogenetically in a novel context, mice exhibited freezing behavior as if they had been exposed to the original training environment. Importantly, hippocampal inactivation did not disrupt the freezing induced by stimulation of the ensemble of tagged RSC neurons, indicating that the RSC can have a functionally independent role from hippocampus in retrieving contextual fear memories. This is consistent with findings described previously, in which RSC inactivation but not hippocampal inactivation produced deficits in sensory preconditioning [48, 50], indicating the RSC but not hippocampus was needed for forming the initial associations between sensory cues. In an application of yet another exciting new technology, RSC neurons have been shown to be active during spatial learning using time-lapse in vivo two-photon imaging [58]. Future studies might use these approaches to compare the activity of RSC neurons during the period between training and recall in order to disambiguate the contribution of RSC to memory storage processes versus the expression of memory.

It will also be important to consider plasticity and neural activity that arises from intra-RSC communication. The intrinsic connectivity of RSC has only recently been described [86] and little is currently known about the nature of information processing within RSC. If RSC is indeed involved in forming, storing, and/or retrieving associations between sensory cues that compose a context, this may be reflected in a strengthening of synapses between RSC neurons. Moreover, RSC is composed of multiple, distinct anatomical subregions [9–11] and only a few studies to date have investigated the functional differences between these areas (e.g., [87–89]). Thus, future research that considers communication and plasticity between these subregions may yield additional insight into the nature of information processing within RSC.

In addition to using technological advances to better understand the contribution of RSC to memory processes, it will be useful to consider behavioral experiments that could further delineate the functions of RSC. For instance, as mentioned previously, existing data indicate that RSC is needed for the successful encoding and retrieval of associations between sensory stimuli (e.g., contextual cues) but not associations between an individual cue (e.g., a tone) and an outcome (e.g., foot shock). However, the studies to date have only considered the effects of RSC manipulations on* recently* acquired cue-outcome associations [27]. Considering that the RSC has a critical role in the retrieval of older (remote) contextual memories, it might also contribute to the retrieval of remote cue-outcome associations. One possibility is that over time, memory for a discrete stimulus like a tone becomes integrated with the memory of the context in which the cue was experienced. Indeed, while learning and performance is often unaffected by a change in context (e.g., [90]), less is known about the impact of a context change following a retention interval (i.e., a remote memory). If cue-outcome associations indeed become contextually controlled over time, the RSC might contribute to the retrieval of remote cue-outcome associations especially considering the crucial role of the RSC in contextual memory.

## 9. Conclusions

Despite the relatively large size of RSC and its integral position in the where/when pathway, its specific contribution to hippocampal-dependent forms of learning and memory is only now beginning to emerge. To date, the bulk of the behavioral evidence supports the idea that RSC is specifically involved in forming and retrieving associations among the neutral stimuli that are present in the environment. Importantly, the role of the RSC is not limited to processing physical stimuli such as visual, auditory, and tactile cues but also extends to temporal cues. The evidence of activity-dependent neuroplastic changes in RSC neurons further supports the view of RSC as a site in which multiple cues are linked together in the service of memory formation, storage, and retrieval. However, additional work is needed to specifically determine if and how RSC contributes differently to recent versus remote memory. Future studies that make use of burgeoning technologies such as optogenetics, chemogenetics, and optical imaging will also be extremely valuable in further delineating the involvement of RSC in storage versus retrieval processes and in defining the precise mechanisms through which RSC binds stimuli together.
